# Introduction: the 12th annual conference of The Australia & New Zealand Academy for Eating Disorders

**DOI:** 10.1186/2050-2974-2-S1-I1

**Published:** 2014-11-24

**Authors:** Anthea Fursland, Jeremy Freeman

**Affiliations:** 1 Centre for Clinical Interventions, Perth, Australia; 2 Australia & New Zealand Academy of Eating Disorders, Sydney, Australia

## 

The 12th annual conference of The Australia & New Zealand Academy for Eating Disorders was held on 22-23 August 2014 at the Esplanade Hotel, Fremantle, Western Australia. 68 Oral papers and 14 Posters were presented following review of the Scientific Committee chaired by Dr. Anthea Fursland and A/Prof Susan Byrne, and are published in this supplement. Over 250 delegates attended the conference.

Keynote talks were by A/Professor Kelly Vitousek, from the University of Hawaii: More similar than strange: *The power of “normal” explanations in the treatment of eating disorders* and Prof. Tracey Wade of Flinders University: *How do genes and the environment work together to increase risk for eating disorders?* The conference also involved two Plenaries, Body Image (Jessica Smith, Phillippa Diedrichs, Beth Shelton and Susan Paxton) and Binge Eating Disorder (Kathleen Chinn, Fiona Sutherland, Emma Dove and Amy Lampard).

Three pre-conference workshops provided a variety of topics. Kelly Vitousek’s workshop focussed on utilising therapist-assisted exposure. Tracey Wade examined the evidence base for eating disorder prevention. Local GP Sue Martin chaired a session where Janice Russell, Terrill Bruere and Andrew Kennedy presented on gastro-intestinal complications, Polycystic Ovarian Syndrome and skeletal health. In addition 12 in-conference workshops addressed a range of clinical and practical themes. The conference concluded with a debate on the proposition that “Eating Disorders are Brain Disorders” featuring Geoff Buckett, Carolyn Costin, Sloane Madden, Jessica Moncrieff-Boyd, Stephanie Wade and Tracey Wade.

